# A Mobile App (MyPeer) Co-Designed With Immigrant Adolescents for Better Sexual and Reproductive Health: Usability Study

**DOI:** 10.2196/81115

**Published:** 2025-12-25

**Authors:** Salima Meherali, Amyna Ismail Rehmani, Mariam Ahmad, Piper Scott-Fiddler, Paula Pinzón-Hernández, Zeba Khan, Sarah Flicker, Philomina Okeke-Ihejirika, Bukola Salami, Eleni Stroulia, Ashley Vandermorris, Josephine Pui-Hing Wong, Wendy V Norman, Shannon D Scott, Sarah Munro

**Affiliations:** 1 Faculty of Nursing College of Health Sciences University of Alberta Edmonton, AB Canada; 2 Department of Obstetrics and Gynecology University of British Columbia Vancouver, BC Canada; 3 Faculty of Environmental and Urban Change York University Toronto, ON Canada; 4 Department of Women’s and Gender Studies Faculty of Arts University of Alberta Edmonton, AB Canada; 5 Department of Community Health Science Cumming School of Medicine University of Calgary Calgary, AB Canada; 6 Faculty of Science University of Alberta Edmonton, AB Canada; 7 Department of Paediatrics University of Toronto Toronto, ON Canada; 8 Daphne Cockwell School of Nursing Toronto Metropolitan University Toronto, ON Canada; 9 Department of Family Practice Faculty of Medicine University of British Columbia Vancouver, BC Canada; 10 Department of Health Systems and Population Health School of Public Health University of Washington Seattle, WA United States

**Keywords:** adolescent health, mHealth, sexual and reproductive health, co-design, usability testing

## Abstract

**Background:**

Adolescents require comprehensive sexual and reproductive health (SRH) education to successfully transition from puberty into adulthood. However, they often experience barriers and challenges while trying to promote their SRH or access SRH services. Such challenges are amplified among youth from migrant backgrounds, who may further be constrained by societal stigmas and cultural taboos regarding SRH. Mobile health interventions have the potential to provide culturally relevant, accessible, and evidence-based SRH educational resources; however, few SRH mobile apps in Canada are co-designed with immigrant youth or meaningfully integrate their voices and lived experiences.

**Objective:**

We aimed to co-design a culturally relevant and evidence-based mobile app with immigrant adolescents to provide accurate SRH resources. In this paper, we present the findings of the usability testing of our SRH mobile app—MyPeer.

**Methods:**

Throughout our study, we used a community-based participatory research approach and implemented the principles of human-centered design to co-design our mobile app. For our usability study, we recruited immigrant adolescents and interest holders working with the target population. Adolescents participated in moderated focus group discussions (FGDs) and interest holders evaluated the app’s quality using the standardized Mobile App Rating Scale (rating components on a scale of 1-5). All FGDs were audio-recorded and later analyzed to implement changes in the app. Mobile App Rating Scale (MARS) scores and responses were analyzed descriptively to evaluate the app’s engagement, functionality, aesthetics, quality of information, and subjective app quality.

**Results:**

Overall, 25 adolescents and 17 interest holders participated in this usability study. We analyzed the findings from the FGDs and categorized them into four categories: (1) navigation and interface, (2) SRH information quality and learning, (3) technical performance, and (4) accessibility and multimedia usability. Adolescents found the app visually appealing and the interface easy to navigate. They appreciated interactive features, such as quizzes, and the presentation of information through various media (eg, videos and infographics). However, they also identified technical issues, such as map glitches and navigation inconsistencies, and requested deeper content on SRH topics. The data from the MARS checklist completed by interest holders were analyzed descriptively. The app received the highest scores in the domains of functionality, with mean scores of 4.3 (performance and navigation); engagement, with mean scores of 3.7 (interest); and aesthetics, with mean scores of 4.1 (graphics) and 3.9 (visual appeal). The lowest rated items were customization, with a mean score of 2.5, and interactivity, with a mean score of 3.1.

**Conclusions:**

Our app—MyPeer—has promising usability and appeal for adolescents looking for SRH information. Incorporating feedback from youth and content experts helped identify both technical refinements and content requirements. Our findings support the app’s potential as a scalable, youth-centered SRH digital tool and emphasize the value of participatory design in youth digital interventions.

## Introduction

Adolescence (ages 10-19 years) is a critical period of development during which individuals develop their identity and lay solid foundations for subsequent phases of life [1]. These foundations include a healthy and comprehensive understanding of their sexual and reproductive health (SRH), including puberty, sexual maturity, relationships, and much more [1]. However, research in the field of adolescent SRH suggests that adolescents do not always have the opportunity or access to the best possible SRH education during this crucial phase of their life [2,3]. In many situations, such access is even more restricted for adolescents from migrant backgrounds due to the stigma surrounding SRH, parental unwillingness to discuss SRH, and inability to seek help due to fear and shame [4,5]. A combination of these factors can result in a lack of medically accurate information and increase the risks of unhealthy behaviors.

With the onset of digital technology, health education has become a promising channel to fill information gaps and potentially change behavior [6,7]. According to Statistics Canada [8], 99.2% of people aged 15-24 years use the internet, and 96% own a smartphone, making digital platforms highly impactful for health education. Mobile health (mHealth) apps provide private, youth-friendly platforms that are accessible on demand, offering trusted information and linking youth to services [9,10]. This is particularly beneficial for designing SRH digital interventions as innovative solutions to address sensitive SRH issues [11]. Research globally suggests that such interventions have been successful in reaching, engaging, and educating adolescents and young adults; in some cases, the rate of preventive testing, delayed sexual activity, and SRH knowledge has been improved [11-13]. Several apps have been developed to promote adolescent SRH; however, there is limited information on their quality and accuracy, and few are specifically designed to meet the needs of adolescents from migrant backgrounds in Canada.

To better understand the range of mobile apps available for adolescent SRH in North America and to support the potential development of a new app tailored for adolescents from migrant backgrounds in Canada, we conducted an environmental scan in 2022 [14]. This scan assessed the quality and usability of these apps and identified 15 relevant apps. Our findings revealed that very few apps offered comprehensive, reliable, and evidence-based information, and most lacked features for customization [14]. Additionally, insights from this scan, combined with extensive research involving adolescents that highlight gaps in cultural adaptation and user engagement strategies, underscore the critical need for evidence-informed, youth-centered design in SRH app development [15]. Therefore, we aimed to co-design an accessible and culturally sensitive knowledge translation resource (ie, mobile app) to provide immigrant adolescents with evidence-based resources to access SRH information. Our paper aims to present the findings of a comprehensive usability study of our mobile app, incorporating feedback from adolescents and SRH experts.

## Methods

### Study Design

**Figure 1:**
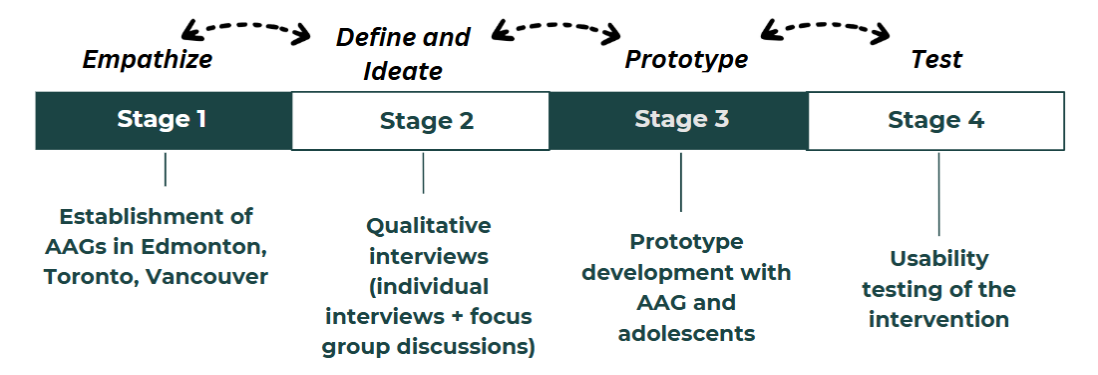
Stages of the co-design project. AAG: adolescent advisory group.

We conducted a multisite study (2023-2024) across 3 Canadian provinces (ie, Alberta, British Columbia, and Ontario), with large immigrant populations. We undertook this study with the purpose of engaging immigrant adolescents in developing, implementing, and evaluating an mHealth app that will deliver evidence-based SRH information to this population. We used a systematic approach to develop a resource that reflects the perceptions and experiences of the target populations as well as to meet their unique needs. Using the principles of human-centered design and community-based participatory research [16], we conducted this study in 4 stages (Figure 1). The protocol for the study and results of the individual stages are published elsewhere [3,15]. This study reports on the findings from stage 4—usability testing.

### Project Stages

#### Stage 1: Empathize

To establish adolescent advisory groups (AAGs), we recruited 26 adolescents (aged 12-19 years) as equal partners in research activities, such as data collection, data analysis, resource development, concurrent feedback, and knowledge mobilization.

#### Stage 2: Define and Ideate

We conducted individual qualitative interviews with immigrant adolescents (n=58) to explore their SRH information needs, access to services, and barriers to promoting their SRH.

#### Stage 3: Prototype

After assessing the SRH information needs of immigrant adolescents and gaps in their knowledge, we developed a prototype for a mobile app specifically catered to their needs.

#### Stage 4: Usability Testing

The multidisciplinary team involved in prototype development included the primary researchers (principal investigator, research coordinator, and youth research assistants), software and app developers from the Department of Computing Science at the University of Alberta, AAG members, and interest holders as content experts. At this stage, we aimed to evaluate whether the designed intervention (prototype) was usable by its targeted end users and to identify errors and issues to make it more user-friendly [10]. This paper reports on the process and outcomes of the usability testing of the MyPeer app prototype.

### Ethical Considerations

We received ethics approval for this project from the University of Alberta Research Ethics Board (August 26, 2021; Pro00113664), the Toronto Metropolitan University Research Ethics Board (April 27, 2022; REB 2022-069), and the University of British Columbia (May 31, 2022; #H22-00395). All participants provided informed consent to participate in the study and were given a gift card (CAD $20 [US $14.5] value) for their time and contribution. Participants were also encouraged to sign a confidentiality agreement to protect the identities of other participants and the experiences shared within discussions.

### Prototype: MyPeer

Our app prototype, MyPeer, is designed to provide adolescents with evidence-based, culturally relevant resources for SRH. This app was co-designed with adolescents from migrant backgrounds, incorporating their unique needs and cultural perspectives. The app provides SRH educational content on 6 SRH priorities (ie, puberty, menstruation, sexually transmitted infections, sexual assault, contraception, and healthy relationships), which were identified and informed through qualitative interviews (stage 2) and AAG design thinking sessions (stage 1). The content was developed by systematically reviewing current literature, evidence-based SRH frameworks (World Health Organization), and national health agency guidelines (Public Health Agency of Canada). We collaborated with SRH experts (eg, clinicians, sexual health consultants, and public health nurses) to validate the content and to ensure its accuracy, and we recommended enhancements to promote its youth friendliness. These experts worked with racialized and migrant populations on issues pertaining to SRH and had practical knowledge about their current challenges. We offered the content through a variety of multimedia formats, such as videos, infographics, text-based information, and animations. The content was developed with an aim to address common cultural stigmas, misconceptions, and knowledge gaps, while promoting positive SRH attitudes and conversations within families and communities. In addition to providing comprehensive SRH content, the app linked adolescents with nearby SRH resources and services (eg, sexual assault centers and birth control clinics), educated them on the latest developments in the field of SRH (via blogs), and promoted engagement through quizzes. Our AAG members contributed to the app’s design and layout, offering suggestions to improve its features and overall appeal. The prototype was made available for testing on both iOS and Android devices.

### Participants and Recruitment

For the usability testing of our prototype, we deemed it beneficial not only to test the app with its intended target audience (ie, adolescents) but also with relevant interest holders (eg, youth advocacy organizations, SRH experts, and health care professionals) working with immigrant adolescents. This approach provided us with a more nuanced understanding of the app’s functionality, acceptability, and relevance within the broader SRH ecosystem. Moreover, involving interest holders in the testing gave us an overview of how digital interventions may be perceived among youth, considering their consistent involvement with this demographic.

We recruited participants from Edmonton, Toronto, and Vancouver between April and December 2024. Our AAG members assisted with recruiting adolescent participants for the app usability testing. We used a multifaceted approach to promote recruitment activities, which included sharing posters via social media, distributing recruitment materials within community networks, email listservs, and youth organizations, and following up with adolescents who participated in our qualitative interviews (phase 2). We used our study’s criteria for inclusion of participants who were either first-generation (born outside of Canada and immigrated to Canada) or second-generation (born in Canada with at least 1 foreign-born parent) immigrants. Additionally, our criteria required that adolescents should be residing in Canada during the time of the study and have access to a mobile device with internet (Android or iOS) to use the mobile app and participate in research activities. We ensured that we had an equal number of participants testing the app on both iOS and Android platforms. We provided all potential participants with an overview of the study, its objectives, the tasks associated with testing, and the time commitment required for each phase of testing. Participants were required to review and sign a consent form and complete a demographic form, which was offered securely through Google Forms.

To recruit interest holders, we contacted relevant interest holders through our network who work with adolescents and have expertise in youth SRH, such as through reproductive health clinics and youth advocacy organizations. We included a diverse range of interest holders, including frontline professionals working with youth (eg, physicians, nurses, and educators) and experts involved in youth-focused initiatives and advocacy efforts (eg, Action Canada).

### Testing Procedures and Measures

We used a multimethod approach to evaluate the usability of the mobile app prototype, incorporating exploratory testing through open-ended focus group discussions (FGDs) and quantitative assessment testing surveys.

#### Testing Measures With Adolescents

We invited adolescents to participate in FGDs (5-6 members each) via Zoom, which lasted for 60-90 minutes. The FGDs moderated by the research team members (SM, MA, and AIR) included open-ended discussions to capture user perceptions, challenges, app performance, and recommendations (see Multimedia Appendix 1). We provided participants with case-based scenarios that involved using various features of the app. They shared feedback on their experience and engaged in discussions about the most effective features and areas for improvement. All the FDGs were audio-recorded for later analysis. After the FGDs, we invited participants to complete a survey rating the app’s attractiveness, user-friendliness, navigation, and usability on a Likert scale, which was offered via Google Forms. It also collected descriptive responses from the participants on their thoughts about the app interface, design, graphics, and suggestions for improvement.

#### Testing Measures With Interest Holders

With the diverse expertise of our interest holder group, we leveraged their insights to thoroughly evaluate the app's quality using the Mobile App Rating Scale (MARS) checklist [17]. MARS is a validated tool that provides a multidimensional, reliable, and flexible app quality assessment criterion, which researchers and academics can effectively use to gather data on the app’s engagement, functionality, aesthetics, quality of information, and subjective app quality [17,18]. We contacted individual interest holders and gave them an overview of the app and the MARS survey. We asked them to review the app content and its features in detail and then complete the survey on their own time. Any concerns or questions regarding the sections of the survey were addressed by the team.

### Data Analysis

#### Quantitative Analysis

Quantitative data from the MARS checklist were analyzed descriptively, and the mean scores were calculated for each of the 5 MARS domains: engagement, functionality, aesthetics, information quality, and subjective quality. Higher mean scores indicated better app quality and perceived usability. Moreover, the demographic characteristics of adolescents were analyzed through descriptive statistics.

#### Qualitative Analysis

We analyzed the qualitative data from the FGDs iteratively, which helped us to modify our questions and expand our understanding of each subsequent FGD. The research team independently reviewed audio transcripts to familiarize themselves with the data and to identify recurrent ideas related to content relevance, app features, navigation, and usability through inductive coding. Categories were iteratively refined and organized into overarching themes. Any discrepancies in the final categorization process were resolved through team discussion to ensure analytic consistency.

## Results

### Participant Characteristics

We recruited 25 adolescents and 17 interest holders for the usability testing. We ensured that our sample included individuals from diverse backgrounds to gather a variety of perceptions and experiences that would help inform the continuous improvement of the app. We collected sociodemographic information from adolescents only, which is presented in Table 1. Of the 25 adolescents, 23 completed the post-FGD survey evaluating the app’s features. The group of interest holders included 5 public health nurses and clinicians, 4 health promotion facilitators (eg, Birth Control Centre), 3 SRH educators, and 5 individuals from youth advocacy organizations (eg, Action Canada and Canadian Advisory of Women Immigrants).

**Table 1:** Demographic characteristics of adolescent participants (N=25).

Characteristics			Values, n (%)
**Sex**			
	Female	19 (76)	
	Male	6 (24)	
**Age (years)**			
	14-15	1 (4)	
	16-17	3 (12)	
	18-20	21 (84)	
**Place of birth**			
	Northern America	13 (52)	
	Sub-Saharan Africa	2 (8)	
	Southern Asia	7 (28)	
	Western Asia	2 (8)	
	Eastern Europe	1 (4)	
**Ethnicity**			
	Asian (South Asian, Southeast Asian, East Asian, and Persian)	17 (68)	
	Black	6 (24)	
	White	2 (8)	
**Current level of education**			
	Grade 10	1 (4)	
	Grade 12	4 (16)	
	Postsecondary school	20 (80)	
**Do you access SRH^a^ services?**			
	Yes	11 (44)	
	No	14 (56)	

^a^SRH: sexual and reproductive health.

### Key Findings

#### Adolescent Perspectives

Adolescents provided detailed feedback on the app’s design, functionality, and user experience. The findings from each FGD were analyzed iteratively to inform the questioning for the next discussion and make necessary changes in the app (eg, technical glitches). We present below the findings from adolescents’ feedback in 4 categories.

#### Navigation and Interface

**Figure 2:**
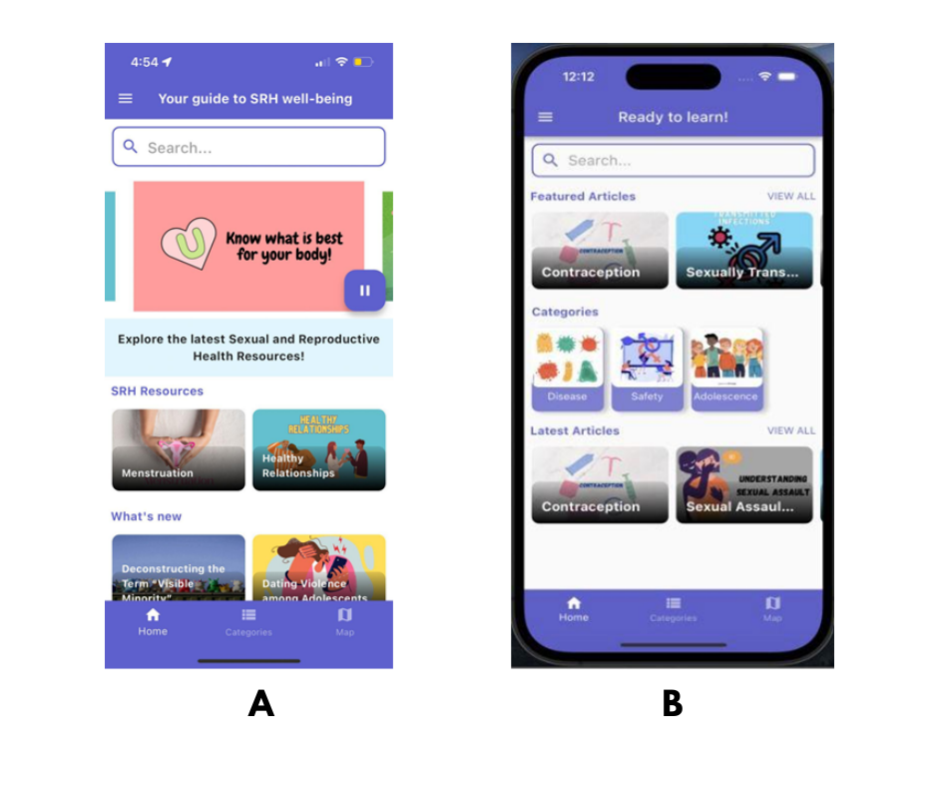
Comparison of the current (A) and previous (B) interfaces of the MyPeer app, highlighting simplified navigation and enhanced visual appeal.

At this stage, it was insightful for us to learn how adolescents perceived the initial design of the app and adjusted to ensure user-friendliness. Many participants appreciated the app’s design and commented on its simplicity and color theme (Figure 2): “The fonts, colors and graphics are probably my favorite thing about the app” [Participant 10].

They liked the display and arrangement of information:

The topics were well arranged, especially the horizontal icons, hence easy to identify. The different bright colors were eye-catching, and also the animations were relatable.

Participant 2

However, several others reported difficulties navigating the app as they found the layout unclear and the content unorganized. Participants emphasized that the home page appeared cluttered and overwhelming, making it difficult to understand how to access desired content. One participant commented that they found the layout confusing:

Having the “categories” button at the bottom confused me into thinking there were more options to choose from, but we had already been shown what the app has to offer on the first page.

Participant 13

Adolescents expressed that it took a lot of exploring to understand the format of the content.

Participants also highlighted how the inconsistent use of fonts, image sizes, and information presentation hindered their ability to intuitively explore the app:

The categories aren’t set up well...the organization can be a lot better. Some tabs look different even though they serve the same purpose.

Participant 8

Another adolescent expressed similar thoughts: “I couldn’t tell where one section ended and another began...the dropdowns made it worse” [Participant 14].

Along with their perceptions, adolescents provided feedback on how certain features could be improved that would make the app more appealing. Participants suggested repositioning the navigation elements (eg, sidebar and top panel) and making the sliding images on the home page clickable: “The sidebar order felt random...maybe sort it based on what’s most useful first” [Participant 3]. Another participant expressed:

It would've been simpler if I liked the sliding images, but assumed they could be clicked on. Not knowing what I could or couldn't click was a little difficult to rummage through as some options were clickable like the categories and quizzes, but the top sliding images were not.

Participant 15

Based on their feedback, we reorganized the navigation elements to present the content more clearly and added the feature to make the sliding images clickable. We also included content within each picture to provide a better understanding of their SRH rights and how they can take care of their bodies.

#### SRH Information Quality and Learning

We inquired about the perceptions of adolescents regarding the SRH information and content presented through different categories and whether the information felt youth-friendly and easy to understand. We emphasized that the selection of content was informed by findings from adolescent interviews and advisory group discussions, and encouraged participants to suggest any additional topics they felt should be included.

Many participants appreciated the SRH topics covered and indicated that these topics were essential for SRH education during adolescence. Adolescents indicated that the information presented throughout the app, including FAQs, quizzes, articles, and videos, was accurate and appropriate. They indicated that the language used was simple and avoided unnecessary jargon, but could be simpler and more engaging. One participant mentioned:

I really like the information that the app provides and the use of color in every section. I would like to see more consistency with the fonts and also making it more reader-friendly.

Participant 11

Some participants inquired about including more expansive explorations of SRH concepts with embedded definitions in quizzes or links to further learning resources. Participants expressed a desire for more in-depth information on specific topics, such as contraception, and recommended including content that addresses the cultural dimensions of these subjects. One participant mentioned:

Some of the content was too intensive and some was lacking information. This mainly applied to the contraceptive section where it goes through every possible form of contraceptive, but didn't really go in depth on how to use them. If I were a new adolescent and reading through it I would be confused by the description of how to use it and how to acquire such things.

Participant 20

Another participant expressed:

I wish there were more diverse stories or even real experiences shared to reflect on. I was glad that there were lots of information available for things like menstruation and contraceptives—which are important! But I was slightly disappointed to see less information about sexual assault or dating violence.

Participant 9

Based on this feedback, we expanded information on topics such as contraception and provided links to additional resources at the bottom of each topic. We also included blogs by our team on topics such as dating violence to raise awareness on these issues and support informed decision-making among users.

Some participants also expressed a desire to see features, such as chatbots or helper chats and discussion forums, embedded within the app, where users can ask questions and get accurate answers or participate in discussions among their peers: “I would like to see like a topic forum where I can search and get like an expert opinion” [Participant 13]. While the idea was appreciated, concerns were raised about the potential harm, such as negative conversations (eg, bullying) and the spread of misinformation through these forums.

Participants shared their feedback regarding the app’s quiz feature and whether this would be a good motivator for app users to continue to improve their knowledge. Many adolescents found the quizzes interactive and engaging and expressed that they challenged them to learn more: “I liked the quizzes because it sort of challenged me to try to retain as much information as possible and made it a more interactive app” [Participant 18].

Participants also reflected on how the quiz section can be improved to ensure it is impactful. They suggested that definitions for difficult terms or acronyms can be presented as a pop-up window or at the bottom of the content.

One adolescent mentioned:

I would want the quiz section to improve because when you get the right answer, it's very short. I have done quizzes in different apps before, and the long explanatory answer was what I liked the most about the other apps.

Participant 17

#### Technical Performance

Throughout our discussions, we found that participants experienced recurrent technical difficulties, particularly those related to the map feature and quiz navigation. Some adolescents commented that their map feature did not automatically detect their location, and they were unable to locate services nearby: “I liked the map feature even though it wasn't working perfectly for me” [Participant 22]. Some adolescents also suggested including a pop-up explaining the purpose of the map and a brief overview of the content within it.

**Figure 3:**
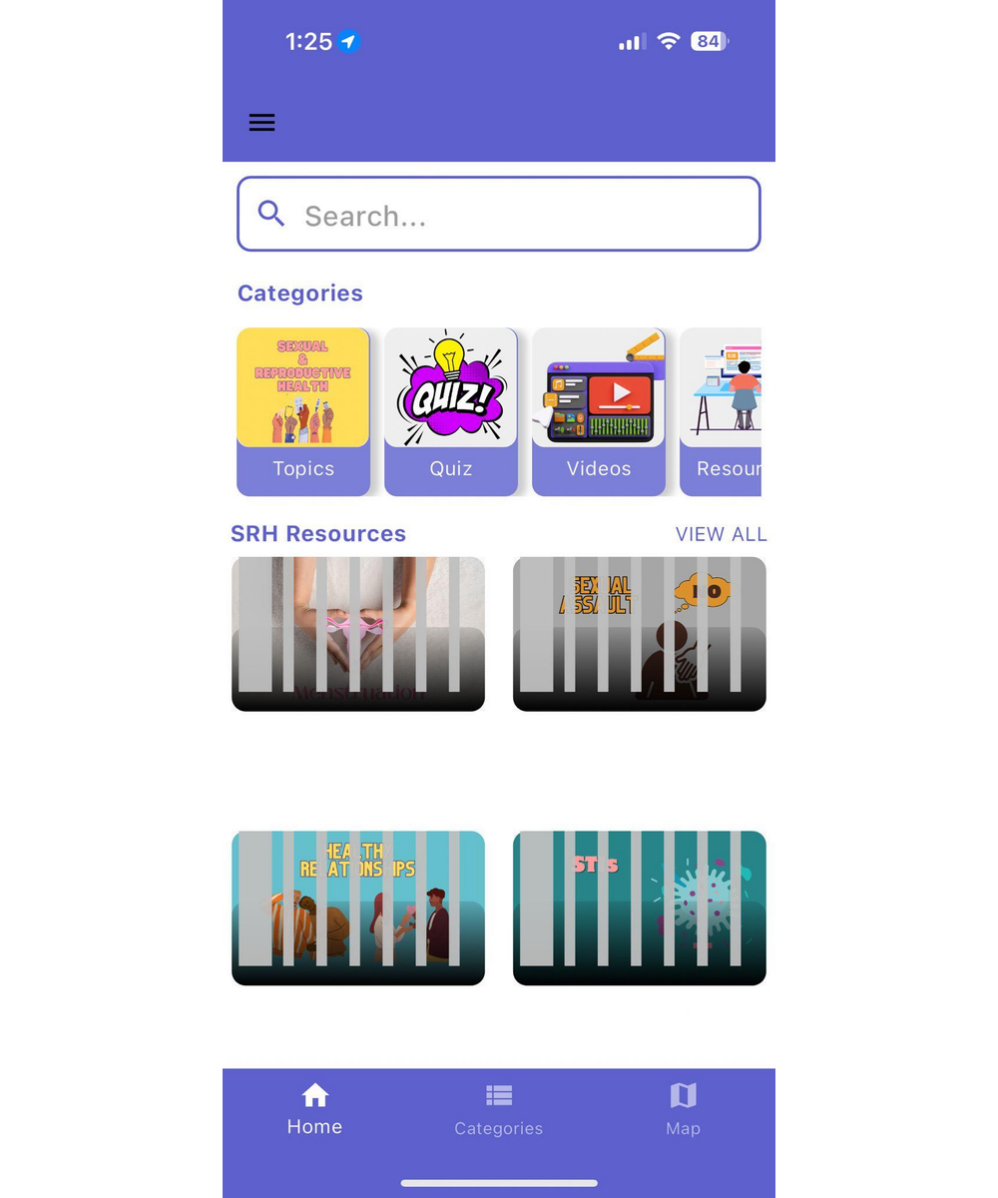
Reported technical glitches during the usability testing of the MyPeer app, including slow loading times and inconsistent navigation.

As adolescents tested various features of the app, we were able to identify several bugs and performance issues that allowed us to highlight areas requiring further improvements (Figure 3). For example, one participant noted that after completing a quiz and returning to the home page, they observed more categories than were originally displayed. This feedback enabled us to identify an underlying coding issue, which was subsequently addressed by our app developer. Similarly, other participants pointed out glitches with the app interface, such as the inability to close the keyboard after performing a search or rapid transitions between quiz questions that left little time to process: “The quizzes still skip questions, which prevents me from answering all the questions” [Participant 24]. Another participant mentioned, “My keyboard popped up randomly when I opened the map or search, and then I had to tap like five times to make it go away” [Participant 16].

We tried to address the challenges regarding the technical performance of the app as soon as we received feedback. This helped us to rectify those errors and test whether the issues were resolved in the subsequent FGD. As we progressed to a new FGD, we also identified some new technical issues that hadn’t been present initially and might have been generated as a result of updates made to the firmware. For example, one participant mentioned that the videos embedded within each category were auto-playing as they opened that category. With the expertise of our app developer, we were able to fix all bugs and restore the app to its optimal performance, which were then not reported in the subsequent FGD.

#### Accessibility and Multimedia Usability

We inquired about adolescents’ views on the information presented through various means (eg, infographics, videos, and pictures) by using case-based scenarios during FGDs. By participating in those activities, adolescents could indicate which resources proved effective and which could be improved. Adolescents emphasized the need for improved accessibility features, such as text-to-speech options, transcripts for videos, and more intuitive media control (eg, full-screen buttons). As one participant mentioned:

I liked the videos, as it makes the content more accessible for those who are more adept at listening. It would be helpful to have a transcript or subtitle to make this information accessible. For the infographics, I would have appreciated being able to click and enlarge the photos.

Participant 5

Many participants commented on the design and limited Zoom functionality of the infographics, citing difficulties reading the font. Aligning with other feedback from participants regarding the consistency of fonts across different categories, we made major improvements in the design of the infographics to ensure their readability and consistency. We also made improvements to the color scheme of the infographics to make it more appealing.

Participants had contrasting views on the way the information within categories was presented. Some adolescents liked how information was categorized and presented under drop-down menus, which made it look less cluttered. However, others did not find manually minimizing the dropdowns appealing. One participant indicated alternatives for drop-down style content:

The drop-down menu for the infographics page makes it hard to access and read the information. It would be helpful if the infographic went to a different page.

Participant 17

While we appreciated the feedback, this feature was not supported by our current app coding framework and was not deemed possible to implement at that stage.

**Figure 4:**
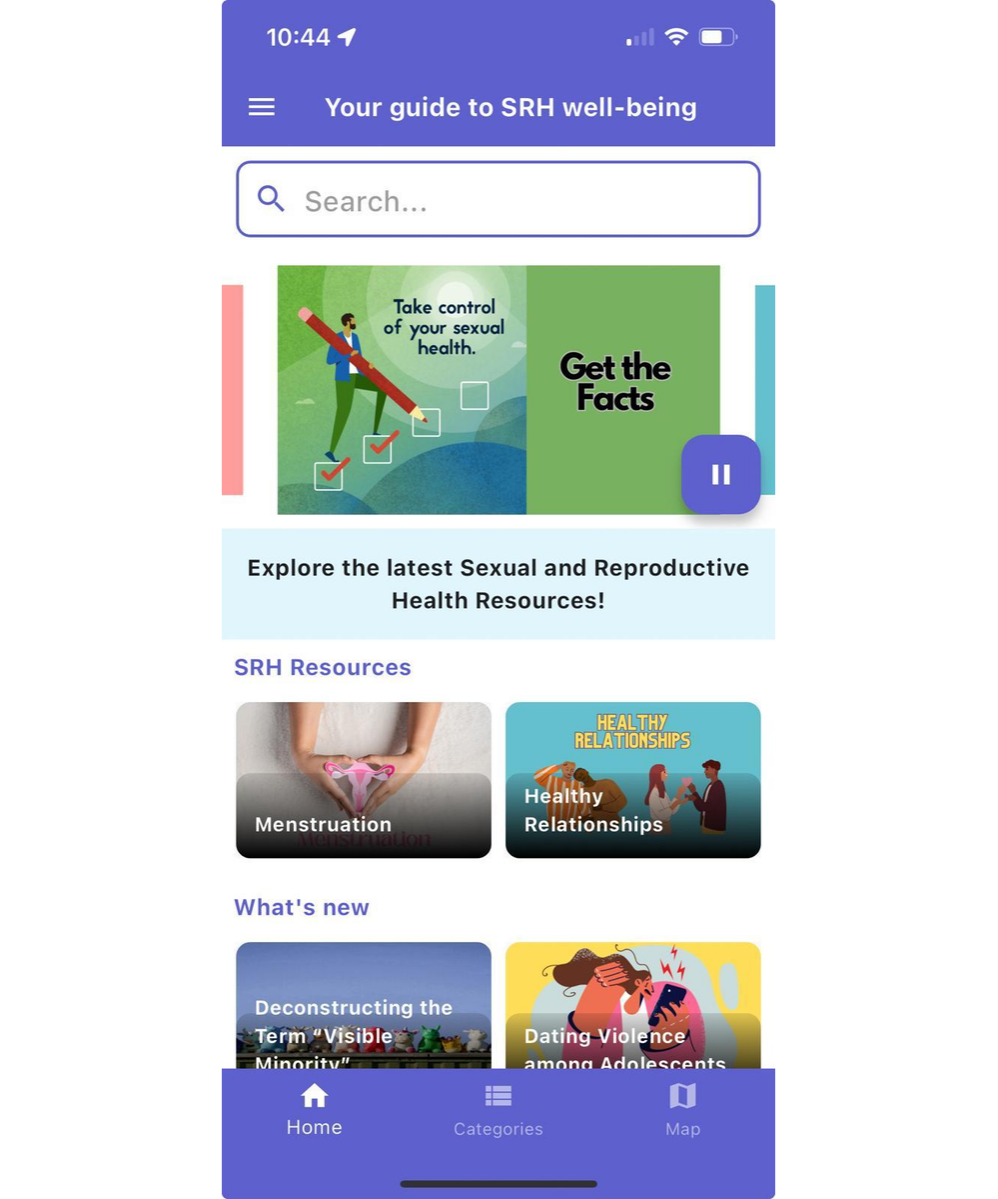
Landing page of the MyPeer app providing adolescents access to educational content, blogs, and navigation to additional resources and services.

Along with the content within categories, we also inquired about the accessibility of other features within the app. Participants commented that they appreciated the maps feature as it distinguished the app from other information that is available online. They also liked including blogs within the content as they can provide more recent and up-to-date information on SRH topics. Initially, the rotating images banner on our home screen did not have a pause feature. After receiving the following feedback, we integrated a pause and play button for the rotating banner (Figure 4):

After a while, it was a bit disorienting to watch the banners keep rotating. It would be helpful to have the ability to pause or click on a banner.

Participant 9

**Figure 5:**
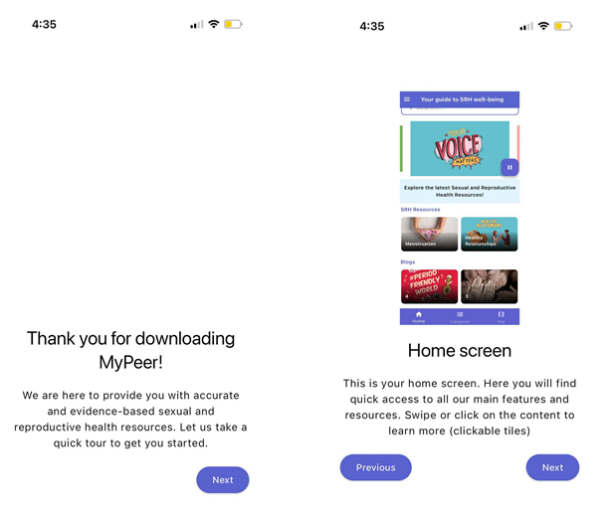
Guided tour of the MyPeer mobile app outlining key features and disclaimers (privacy and security).

A couple of participants mentioned that it would be beneficial to include a tour-style guide for users when they first download the app to avoid confusion for new users. We implemented this feedback and included a walk-through of different features of the app, which was well received by consecutive FGDs (Figure 5). Some participants also pointed out that not all adolescents would like to register and provide their information; such individuals, in their view, should be able to access the content without registering. We considered this feedback and added the option to access content without registering.

#### Stakeholder Assessment Using the MARS Checklist

A total of 17 interest holders, including sexual health educators, researchers, academics, and health professionals, completed the MARS checklist. We conducted this stage of this study after making most of the changes identified through adolescents’ feedback. According to the checklist, their feedback was assessed across four domains: (1) engagement, (2) functionality, (3) aesthetics, and (4) information quality.

#### Engagement

Most interest holders indicated that the app was moderately entertaining, with 29% (5/17) indicating that it was highly entertaining (Table 2). The interactive quizzes, videos, and articles were appreciated for their potential to maintain user engagement and promote learning. Among the participants, 59% (10/17) of interest holders agreed that the app was moderately interesting and would keep users engaged for some time. Some interest holders indicated that the lack of more gamification strategies might limit long-term retention.

**Table 2:** Mean scores for the engagement domain of the Mobile App Rating Scale checklist.

Item		Mean score
**Entertainment**		
	Is the app fun and entertaining to use?	3.6
**Interest**		
	Is the app interesting to use?	3.7
**Customization**		
	Does it provide or retain necessary settings and preferences?	2.5
**Interactivity**		
	Does it allow user input and feedback?	3.1
**Target group**		
	Is the content appropriate for adolescents?	3.9

#### Functionality

This domain received high ratings, with 53% (9/17) of participants reporting that the app’s performance was perfect, while 29% (5/17) reported minor problems, and 18% (3/17) of interest holders reported some technical problems. These issues were primarily due to known bugs and technical glitches, which were identified during the adolescent FGDs as well. Despite these issues, the app was considered functional in its core delivery and was ranked highly for its ease of navigation (Table 3).

**Table 3:** Mean scores for the functionality domain of the Mobile App Rating Scale checklist.

Item			Mean score
**Performance**			
	How accurately or fast do the app features work?	4.3	
**Ease of use**			
	How easy is it to learn to use the app?	4.2	
**Navigation**			
	Is moving between screens logical or accurate or uninterrupted?	4.3	
**Gestural design**			
	Are interactions consistent and intuitive?	4.2	

#### Aesthetics

The majority of interest holders rated the app’s graphics and aesthetics as high quality, with 41% (7/17) describing the visual appeal as “very attractive” and an additional 24% (4/17) indicating a high level of appeal. Interest holders noted some inconsistencies in the graphics and visual design, and a few also reported issues with icon and content sizing on smaller screens (Table 4).

**Table 4:** Mean scores for the aesthetics domain of the Mobile App Rating Scale checklist.

Item		Mean score
**Layout**		
	Are the arrangement and size of icons and content appropriate?	3.8
**Graphics**		
	How high is the quality of graphics?	4.1
**Visual appeal**		
	How good does the app look?	3.9

#### Information

The majority of the interest holders found the SRH content to be credible, evidence-based, and appropriate for the target audience, with 59% (10/17) of interest holders indicating that the quality of information was highly relevant, coherent, and correct. Moreover, 47% (8/17) of interest holders reported that the information covered was comprehensive and concise and included links to further information and resources (Table 5). However, they suggested increasing comprehensiveness by providing more elaborate explanations, especially in areas where adolescents may seek additional clarification.

**Table 5:** Mean scores for the information domain of the Mobile App Rating Scale checklist.

Item		Mean score
**Accuracy of app description**		
	Does the app contain what is described?	4.3
**Quality of information**		
	Is the app content correct, well-written, and relevant?	4.4
**Quantity of information**		
	Is the extent of coverage within scope?	4.2
**Visual information**		
	Is the visual explanation clear and logical?	4.3
**Credibility**		
	Does the app come from a legitimate source?	3.8

## Discussion

### Principal Findings

Our study evaluated the usability and perceived effectiveness of our mobile app “MyPeer,” co-designed with immigrant youth to disseminate SRH educational content and raise awareness about existing services. We found that our app was well-received among adolescents and interest holders alike, with feedback and suggestions to improve its user-friendliness, content range, technical issues, and interactive features. By integrating a participatory approach at all stages, our process and findings provide essential insights into developing digital health interventions with youth.

Our content and app development were grounded and centered around adolescent perspectives and experiences, with adolescents involved at all stages through a participatory approach [17], allowing them to take ownership of the process and co-design an intervention that is tailored to their needs. A systematic review of SRH apps recommends that, for SRH apps to be impactful, they should not only be of high-quality design and have relevant information but also be tailored to their target group and well-tested with real users [19]. Through FGDs and individualized testing, we provided participants with the opportunity to critically analyze the app (eg, case-based scenarios) along with using the various features to their best potential. We made efforts to integrate their feedback into the design and content of the app, considering the diverse range of thoughts and perceptions among participants. Some features and suggestions were deemed not feasible to implement (eg, helper chat and discussion forum); however, we tried to find alternative solutions to address those unique ideas. We also asked participants in each FGD to express their thoughts on different features through various modalities (eg, whiteboard activities and surveys). Using various feedback mechanisms enabled us to receive feedback based on each participant’s strengths and weaknesses while ensuring that no participant was left unheard. Martin et al [20] suggest that offering alternative ways to participants with diverse capabilities and strengths enhances the chance for effective technological development by acquiring valuable data from a population with high intra- and intervariability.

Participants initially found the app a bit too simple but easy to use. However, with multiple rounds of feedback and changes, they reported the app’s design to be attractive and visually appealing. The variety of content delivery approaches (eg, videos and infographics) improved the user’s motivation to engage with the app. Studies on youth-centered digital interventions have echoed similar findings, emphasizing the importance of multiple formats of information delivery to cater to youth at different cognitive levels and learning styles [10,12]. The quiz feature was appreciated for its interactive nature, with participants indicating enhancing the questions and adding more detailed explanations. Gamification within mobile apps has been seen to improve motivation, comprehension, and long-term retention of health-related information in various studies [11,20]. Overall, participants believed that the app was an effective resource to improve SRH education and knowledge among youth. To enhance the user experience, participants recommended addressing visual inconsistencies and technical glitches (eg, map calibration errors and unintended category duplications) within the app, which highlighted the importance of robust quality assurance during app development. Similar challenges have been identified in other usability studies with adolescent-centered apps, emphasizing the value of iterative testing with end users throughout the development process and considerations for sustainability of mobile technology [21,22].

A recurring theme within our findings was the desire among adolescents for more comprehensive and contextually relevant SRH information, with specific suggestions for including detailed content on topics such as contraception, aspects of sexual relationships, and cultural aspects of SRH. This is consistent with findings in similar studies where authors argue that the effectiveness of SRH interventions relies not only on the presentation of biological facts but also on the social, emotional, and cultural aspects of sexuality, which is meaningful to youth [9,12,19]. Another important consideration with youth-centered digital interventions is the need for user privacy and data security [23,24]. Many users expressed making the content accessible for individuals not willing to register, and to include statements on how their data will be used and stored, which will enhance user trust and the legitimacy of the app. This may also be connected to social stigma and negative judgment that youth experience while accessing sexual health resources and care.

### Stakeholder Feedback and Engagement

The feedback that we gathered from interest holders using the MARS checklist gave us valuable external insights and validation of the app’s quality, design, functionality, and usability. Including interest holders and field experts in app testing has been recommended to obtain targeted data on the intended population, gain multidisciplinary perspectives, and assess the provider’s perceptions on the perceived impact of such interventions [24]. Most interest holders rated the app’s visual appeal and engagement to be high, along with strong agreement on the accurate and evidence-based nature of the app’s content. Their observation reinforces the value of co-designing digital health interventions with youth and SRH experts, which is suggested by the World Health Organization for developing youth-friendly interventions [10]. However, some similar concerns—as expressed by adolescents—were raised regarding the inconsistencies within the graphics, app design, and content presentation on different screen sizes. This highlights the need for responsive design approaches in assuring that the app runs smoothly across a variety of devices and platforms [25,26].

### Limitations

Although our study implemented co-design and participatory approaches at each step, we acknowledge a range of limitations. First, the sample size may not be representative of the entire adolescent population from migrant backgrounds in Canada. It is possible that certain cultural or ethnic groups' views may not have been expressed. Moreover, the limited number of interest holders may not have captured the full range of institutional perspectives. Second, although the data and feedback we received from the participants were rich, longer-term data on behavior change or knowledge retention were not collected, which could have provided insights into the app’s effectiveness in increasing SRH knowledge. Moreover, we initially aimed to offer the MARS checklist to adolescents as well, so we can have a better understanding and quantitative data on the app’s usability. However, very few adolescents completed the checklist, and the data were not sufficient for analysis. We, therefore, incorporated the various domains of the checklist throughout our qualitative discussions. Third, although our instructions requested that participants use the app and review all features through various approaches for a period of time, we are unable to determine if each participant gave the same thought and attention to each aspect of the app. To mitigate this risk, we applied measures to encourage participants to use the app routinely through daily email reminders, a detailed checklist, and timely feedback. We ensured that each participant in the FGD had an opportunity to use the app and a chance to express their concerns.

### Future Implications

Our findings suggest that integrating comprehensive SRH education and culturally sensitive content can enhance the educational value of SRH apps for immigrant adolescents. Addressing interface issues and technical glitches is important to maintain user engagement and motivation to continue using the app. Moreover, it is essential that adolescents know how their data is being used and protected, considering the sensitive nature of app content and the need for privacy. To evaluate the app’s effectiveness in improving SRH knowledge, future research is needed to assess the impact of the app on knowledge, attitudes, and behaviors through longitudinal designs or randomized controlled trials. Our future research will focus on conducting an effectiveness trial to evaluate the app’s impact on the knowledge, attitudes, and behaviors of immigrant adolescents toward their SRH. We will also work to continuously update and evolve our content based on the latest evidence-based resources and guidelines, and add further content identified through the usability study, which is inclusive and appropriate to diverse populations. For this purpose, we will form further partnerships with youth and community-based organizations to evaluate how we can benefit from our respective work with adolescents collaboratively and engage in knowledge-sharing initiatives.

### Conclusions

Our findings demonstrate that a co-designed, youth-friendly mobile app (MyPeer) can be impactful in addressing stigma, misconceptions, and barriers to access SRH information for adolescents from migrant backgrounds. Through a co-design and participatory approach, we gathered feedback from adolescents and interest holders on the design, functionality, and usability of our mobile app. Our app rated highly in its aesthetics, navigation, and design. The content was also appreciated for its accuracy and was deemed evidence-based by SRH experts. We received feedback and suggestions to enhance the breadth and depth of SRH content, address technical problems, and resolve inconsistencies related to graphics and design. Our findings suggest that immigrant adolescents need clear, evidence-based information offered through a variety of mediums. Beyond its acceptability and perceived effectiveness, our process provides valuable insights into incorporating youth voices within digital health interventions, indicating the importance of participatory design in promoting relevance, usability, and impact of such interventions. Our findings have broader implications for researchers, practitioners, and policymakers exploring the development of inclusive, evidence-based, youth-centered digital interventions. Health educational apps developed for youth to support their health and well-being should be cognizant of their perspectives and their preference to receive educational information.

## Appendix

Multimedia Appendix 1 Focus group facilitation guide.

## Abbreviations

AAG: adolescent advisory group
FGD: focus group discussion
MARS: Mobile App Rating Scale
mHealth: mobile health
SRH: sexual and reproductive health

## Data Availability

The datasets used or analyzed during the study are available from the corresponding author upon reasonable request.

## References

[1] (2019). Adolescent health. World Health Organization.

[2] Langat EC, Mohiddin A, Kidere F, Omar A, Akuno J, Naanyu V, Temmerman M (2024). Challenges and opportunities for improving access to adolescent and youth sexual and reproductive health services and information in the coastal counties of Kenya: a qualitative study. BMC Public Health.

[3] Meherali S, Rehmani AI, Ahmad M, Kauser S, Scott Fiddler P, Pinzon-Hernandez P, Khan Z, Flicker S, Okeke-Ihejirika P, Salami B, Stroulia E, Vandermorris A, Wong J, Norman W, Scott S, Munro S (2024). Between cultures and traditions: a qualitative investigation of sexual and reproductive health experiences of immigrant adolescents in Canada. Cult Health Sex.

[4] Corley AG, Sprockett A, Montagu D, Chakraborty NM (2022). Exploring and monitoring privacy, confidentiality, and provider bias in sexual and reproductive health service provision to young people: a narrative review. Int J Environ Res Public Health.

[5] Sidamo N, Kerbo A, Gidebo K, Wado YD (2024). Exploring barriers to accessing adolescents sexual and reproductive health services in South Ethiopia Regional State: a phenomenological study using Levesque's framework. Adolesc Health Med Ther.

[6] Brayboy LM, McCoy K, Thamotharan S, Zhu E, Gil G, Houck C (2018). The use of technology in the sexual health education especially among minority adolescent girls in the United States. Curr Opin Obstet Gynecol.

[7] Dennison L, Morrison L, Conway G, Yardley L (2013). Opportunities and challenges for smartphone applications in supporting health behavior change: qualitative study. J Med Internet Res.

[8] (2024). A data snapshot of Canadian youth and technology. Statistics Canada.

[9] Ippoliti NB, L'Engle K (2017). Meet us on the phone: mobile phone programs for adolescent sexual and reproductive health in low-to-middle income countries. Reprod Health.

[10] (2021). Youth-centered digital health interventions: a framework for planning, developing and implementing solutions with and for young people. World Health Organization.

[11] Tebb K (2024). Leveraging serious video games to transform HIV prevention and care for adolescents and young adults: the case for playtest!. J Adolesc Health.

[12] Guse K, Levine D, Martins S, Lira A, Gaarde J, Westmorland W, Gilliam M (2012). Interventions using new digital media to improve adolescent sexual health: a systematic review. J Adolesc Health.

[13] Ibitoye M, Lappen H, Freeman R, Jordan AE, Aronson ID (2021). Technology-based interventions to increase point-of-care HIV testing and linkage to care among youth in the US: a systematic review. AIDS Behav.

[14] Benoit JRA, Louie-Poon S, Kauser S, Meherali S (2022). Promoting adolescent sexual and reproductive health in North America using free mobile apps: environmental scan. JMIR Pediatr Parent.

[15] Meherali S, Munro S, Puinean G, Salami B, Wong JP, Vandermorris A, Benoit JRA, Flicker S, Okeke-Ihejirika P, Stroulia E, Norman WV, Scott SD (2023). Co-designing a sexual health app with immigrant adolescents: protocol for a qualitative community-based participatory action research study. JMIR Res Protoc.

[16] Chen E, Leos C, Kowitt SD, Moracco KE (2019). Enhancing community-based participatory research through human-centered design strategies. Health Promot Pract.

[17] Stoyanov SR, Hides L, Kavanagh DJ, Zelenko O, Tjondronegoro D, Mani M (2015). Mobile app rating scale: a new tool for assessing the quality of health mobile apps. JMIR Mhealth Uhealth.

[18] Terhorst Y, Philippi P, Sander LB, Schultchen D, Paganini S, Bardus M, Santo K, Knitza J, Machado GC, Schoeppe S, Bauereiß N, Portenhauser A, Domhardt M, Walter B, Krusche M, Baumeister H, Messner E (2020). Validation of the Mobile Application Rating Scale (MARS). PLoS One.

[19] Muehlmann M, Tomczyk S (2023). Mobile apps for sexual and reproductive health education: a systematic review and quality assessment. Curr Sex Health Rep.

[20] Martin A, Caon M, Adorni F, Andreoni G, Ascolese A, Atkinson S, Bul K, Carrion C, Castell C, Ciociola V, Condon L, Espallargues M, Hanley J, Jesuthasan N, Lafortuna CL, Lang A, Prinelli F, Puidomenech Puig E, Tabozzi SA, McKinstry B (2020). A mobile phone intervention to improve obesity-related health behaviors of adolescents across Europe: iterative co-design and feasibility study. JMIR Mhealth Uhealth.

[21] Fedele DA, Cushing CC, Fritz A, Amaro CM, Ortega A (2017). Mobile health interventions for improving health outcomes in youth: a meta-analysis. JAMA Pediatr.

[22] Shikako K, Mogo ERI, Grand-Maison V, Simpson R, Pritchard-Wiart L, Majnemer A, Jooay App Research Group (2021). Designing user-centered mobile health initiatives to promote healthy behaviors for children with disabilities: development and usability study. JMIR Form Res.

[23] Pretorius C, Chambers D, Coyle D (2019). Young people's online help-seeking and mental health difficulties: systematic narrative review. J Med Internet Res.

[24] Seretlo RJ, Smuts H, Mokgatle MM (2024). Development of an mHealth app by experts for queer individuals' sexual-reproductive health care services and needs: nominal group technique study. JMIR Form Res.

[25] Durgekar SR, Rahman SA, Naik SR, Kanchan SS, Srinivasan G (2024). A review paper on design and experience of mobile applications. EAI Endorsed Scal Inf Syst.

[26] Tomlinson M, Rotheram-Borus MJ, Swartz L, Tsai AC (2013). Scaling up mHealth: where is the evidence?. PLoS Med.

