# Supplementary material for: A Mobile App (MyPeer) Co-Designed With Immigrant Adolescents for Better Sexual and Reproductive Health: Usability Study
Source: JMIR Form Res. 2025 Dec 25;9:e81115. doi: 10.2196/81115 (PMC12784138; doi:10.2196/81115)
Supplement: Multimedia Appendix 1 [file formative_v9i1e81115_app1.docx]

**Focused-Group Facilitation Guide**

**1. Introduction and Ground Rules**

- Welcome participants and thank them for joining.
- Confirm participants consent form submission to proceed.
- Introduce the research team and purpose of the study.
- Explain confidentiality, voluntary participation, and audio recording.
- Encourage open and honest sharing, no right or wrong answers.

**2. Time to explore the app:**

Participants will be given 10-15 minutes to explore the various features of the app through case-based scenarios.

**3. Warm-Up Questions**

- Can you tell us a bit about your general experience using health or educational apps?
- What was your first impression when you opened the MyPeer app?

**4. Usability and Navigation**

- How easy or difficult was it to navigate the app?
- Were the icons, menus, and labels clear?
- Did you experience any technical issues (e.g., loading, login, or scrolling problems)?
- How would you describe the overall design and layout of the app?

**5. Content and Relevance**

- What did you think about the information presented in the app?
- Was the content easy to understand and relevant to your needs?
- Were the topics covered (e.g., puberty, consent, contraception, STIs, healthy relationships) useful or missing anything important?
- Did the app reflect diverse experiences, cultures, and backgrounds appropriately?
- Do you think the app could help adolescents make informed health decisions or seek help when needed?

**6. Engagement and Aesthetics**

- How engaging did you find the app (e.g., visuals, colors, videos, quizzes)?
- Which parts did you find most interesting or motivating to explore?
- Was there anything you found confusing, boring, or overwhelming?

**7. Recommendations for Improvement**

- What changes would make the app easier or more enjoyable to use?
- Are there any features or topics you would like to see added?
- How could we make the app more accessible or appealing for different groups of adolescents (e.g., newcomers, youth with low literacy)?

**Closing**

Is there anything else you would like to share about your experience with the app?

Thank participants for their time and valuable insights.
